# Weedy and seedy: the rapid evolution of life-history characteristics in an introduced daisy

**DOI:** 10.1093/aobpla/plac038

**Published:** 2022-08-18

**Authors:** Claire R Brandenburger, Ben Maslen, William B Sherwin, Angela T Moles

**Affiliations:** Evolution & Ecology Research Centre, School of Biological, Earth and Environmental Sciences, UNSW Sydney, Sydney, NSW 2052, Australia; Mark Wainwright Analytical Centre, UNSW Sydney, Sydney, NSW 2052, Australia; Evolution & Ecology Research Centre, School of Biological, Earth and Environmental Sciences, UNSW Sydney, Sydney, NSW 2052, Australia; Evolution & Ecology Research Centre, School of Biological, Earth and Environmental Sciences, UNSW Sydney, Sydney, NSW 2052, Australia

**Keywords:** Germination, introduced plant, life history, plant traits, rapid evolution, reproductive output, seed mass, survival

## Abstract

Despite the importance of life-history characteristics in determining a species’ success, we still lack basic information about some fundamental life-history elements found across the life cycle of introduced plants. Our study assesses rapid evolutionary divergence in life-history characteristics of the beach daisy *Arctotheca populifolia* by comparing introduced Australian and source South African plants and measuring eight key variables including seed mass, germination, reproductive output and survival. This is the first study that compares the life history of an introduced plant species with its single original source population, providing a precise and powerful method for detecting evolutionary divergence. We found that introduced *A. populifolia* has evolved a suite of weedy life-history characteristics in less than 90 years: the introduced plants use a live-fast die-young strategy of germination and survival and produce significantly more inflorescences and more seeds that germinate faster. This knowledge adds to the remarkable data that we already have on the rapid evolutionary divergence occurring in the morphology, physiology and defence of this introduced plant and highlights the speed and scope of evolutionary divergence possible in plants. To fully understand and manage the future of our plant species, we must consider their potential for ongoing change in key aspects of life history.

## Teaser

Hide How fast could an introduced plant adapt some of its fundamental life-history characteristics to succeed on a new continent an ocean away? We found that in less than 90 years after arriving in Australia, the South African beach daisy has rapidly evolved to become weedier and seedier than its parent population. The introduced Australian plants use a live-fast die-young strategy of germination and survival, and produce significantly more inflorescences and more seeds that germinate faster. In addition, this is the first study that compares the life history of an introduced plant species with its single original source population, providing a precise and powerful method for detecting evolutionary change.

## Introduction

By moving species beyond their natural dispersal limits, humans have unintentionally set up a global experiment in ecology and evolution. New selective pressures in the introduced range can lead to rapid evolutionary changes in introduced plant and animal species (Lee 2002; Stockwell *et al.* 2003; Phillips *et al.* 2006). There have been several examples of introduced plant populations undergoing dramatic changes in morphology (Daehler and Strong 1997; Buswell *et al.* 2011; Brandenburger *et al.* 2019b) and there have been a vast number of studies of enemy release and subsequent changes in growth and defence traits in plants (e.g. evolution of increased competitive ability: EICA; Bossdorf *et al.* 2005; Colautti *et al.* 2009). However, there is surprisingly little information about basic elements of fitness and life history such as germination and survival, and no study to our knowledge that compares the life history of an introduced plant species with its single original source population. Thus, the overarching aim of our study was to fill this gap in our knowledge by assessing the extent of possible evolutionary divergence in the life-history characteristics of introduced *Arctotheca populifolia*. We did this by comparing introduced and source plants and measuring eight key variables including seed mass, germination, reproductive output and survival.

The South African beach daisy *A. populifolia* (Asteraceae) was introduced to Australia in the 1930s, and has since spread and become established along the entire southern half of Australia’s east coast (AVH database 2022). We used common-environment experiments to compare the native and introduced plants but with a novel key addition: instead of comparing introduced plants to plants from across the native range, as is usually done in this type of study, we compared introduced *A. populifolia* plants to their actual parent population. Comparing introduced plants to native plants from across a broad range can introduce variation which can confound the assessment of evolutionary change. Colautti *et al.* (2009) analysed 32 comparisons of native and introduced populations in common-environment experiments and found that among-population variation due to geographic clines was so substantial that when included in the analyses, it could alter the significance, magnitude or even direction of some differences between native and introduced populations. Using the actual source population provides an accurate point of reference against which to assess what changes have taken place and provides a powerful and precise test of the evolutionary divergence possible since introduction. We assessed eight response variables for our investigation, some of which are traits (*sensu*Violle *et al.* 2007), while others, such as germination rate and seedling survival, are key aspects of life-history strategy.

Seed mass is a fundamental trait that correlates with the timing of germination, initial seedling size, seedling establishment and seedling survival (Harper *et al.* 1970; Jakobsson and Eriksson 2000; Leishman *et al.* 2000; Moles and Leishman 2008). Theory suggests that if introduced plants experience enemy release, they may allocate resources from defence to increased growth and/or reproduction (Keane and Crawley 2002), with one possible outcome being the production of larger seeds in the introduced range (Daws *et al.* 2007). Therefore, we predicted that introduced *A. populifolia* would have larger seeds than would source *A. populifolia*.

Germination is a critical stage in the life of any plant, with germination behaviour affecting establishment and survival (Crawley 1997). However, the first study comparing the germination strategies of a species in its introduced and native ranges was carried out relatively recently (Hierro *et al.* 2009). These authors found that the germination strategy of *Centaurea solstitialis* (Asteraceae) in either range was affected by the degree of climatic risk experienced by the plants in their early stage of development, with variation in rainfall after germination being a critical factor selecting for delayed germination. Since *A. populifolia* flowers sporadically all year round (SANBI database 2017) and can be subject to several desiccatory stresses including exposure to high temperatures, high light intensity and wind (Hesp 1991), we would also expect delayed germination under conditions of increased climatic risk. However, because introduced *A. populifolia* plants in Australia experience rainfall that is two to three times higher throughout the year than in the native range, especially in the hotter summer months (Brandenburger *et al.* 2019b), we predicted that the reduction in climatic risk in the introduced range would select for seeds that germinate with less delay and at a higher percentage than source seeds. These predictions align with a recent review of species in their introduced and native ranges which found a tendency for introduced plants to germinate earlier, at greater percentages and over a wider range of germination conditions than plants in the native range (Gioria and Pyšek 2017).

The establishment, persistence and spread of any species are critically dependent on its reproductive output. In the case of introduced species this is especially crucial—if reproductive output is low and the founding population is small, then the introduced species may fail to establish in a new range (Sakai *et al.* 2001). According to the EICA hypothesis (Blossey and Notzold 1995), plants introduced to in a new range should be able to allocate more resources to reproduction because they require fewer resources for defence. However, support for EICA has been mixed. Some studies report that introduced species have the same amount of reproductive output in their home range as in their introduced range (Maron *et al.* 2004; Mason *et al.* 2008; Felker-Quinn *et al.* 2013). However, other studies (Willis and Blossey 1999; Blair and Wolfe 2004; Stastny *et al.* 2005), including a meta-analysis investigating 36 plant species in native and introduced ranges (Hawkes 2007), report that introduced plants show increased reproductive output in their new ranges. Therefore, we predicted that introduced *A. populifolia* would have a greater reproductive output than source *A. populifolia* would. Since a previous study found no evidence for higher vegetative reproduction in the introduced populations (no difference in total biomass between introduced and source plants; Brandenburger *et al.* 2019b), we predicted that the greater reproductive output would be allocated to sexual as opposed to clonal reproduction.

Ever since Baker described his traits of the ideal weed (1965, 1974), introduced plants have been associated with a live-fast, die-young strategy. Recent evidence of this strategy has been provided by meta-analyses reporting higher growth rates in introduced plants (van Kleunen *et al.* 2010; Felker-Quinn *et al.* 2013), and a previous study on *A. populifolia* which found that the introduced Australian plants grow longer than the source South African plants with the same amount of biomass, indicating a faster growth rate (Brandenburger *et al.* 2019b). Therefore, our final prediction was that the introduced plants would have a lower rate of survival to the end of the year-long experiment than would the South African source plants.

In summary, our hypotheses were that compared to source plants, introduced plants would have:

1. Larger seeds;
2. A higher per cent of germination with less delay;
3. Greater reproductive output; and
4. Lower rates of survival.

## Methods

In previous work, we used microsatellite data to determine which population of South African *A. populifolia* was most likely to be the source for the *A. populifolia* in eastern Australia (Brandenburger *et al.* 2019b). By sampling populations spanning the whole native range of *A. populifolia,* we found that the population in Arniston is at least 10^99^ times more likely to be the source population for this introduction of *A. populifolia* than any other South African population studied (Brandenburger *et al.* 2019b). The 10^99^-fold difference is in fact conservative (i.e. probably an underestimate). The assessment has nothing to do with mutation rates but is based on the log of odds for the South African location that is genetically most similar to Australia, versus competing South African sources, with a deliberate change to make the test conservative (i.e. making the competitor locations genetically more similar to Australia; for details, see https://dx.doi.org/10.6084/m9.figshare.c.4392740). Therefore, we set up a common-environment glasshouse experiment using seeds collected from Arniston, South Africa and from four populations spanning 600 km of the introduced range in Australia. Comparing introduced *A. populifolia* plants to their actual source population in Arniston provides a precise and powerful tool to measure evolutionary divergence in life-history characteristics since introduction and is a method that has been used in previous studies of the same introduction (Brandenburger *et al.* 2019a, b, 2020). Since these three previous studies showed that 29/30 traits and life-history characteristics do not differ significantly among the Australian populations **[see****Supporting Information—Appendix S1****]**, we adopted the same method of treating the four Australian populations as a group in our analyses. All plants were grown under standard glasshouse conditions at the University of New South Wales, Sydney, Australia. Temperatures were controlled between 10 °C and 25 °C; daily watering occurred with automatic drippers at 5 pm, as well as 9 am in the seedling stages; and all plants grew in soil with the same composition of river sand, cocopeat and nutrients. For further experimental details, see Brandenburger *et al.* (2019b).

The amount of trait variation observed between different genotypes of a species can vary according to the environmental conditions under which the populations are grown (G × E interactions; Pigliucci 2001). The conditions in our common garden experiment are not characteristic of either the native or introduced ranges for *A. populifolia*. Future researchers might attempt a reciprocal transplant experiment, growing plants from both introduced and native populations in both Australia and South Africa. However, such an experiment would be logistically challenging (in addition to the time and expense required to work at field sites over 11 000 km apart, we would expect survival under natural conditions to be low), and even if it was permissible to plant imported seeds from a different country in natural ecosystems, such an experiment would be ethically questionable.

The environment in which a plant grows can affect several traits of its offspring, despite the genetic make-up of the plant (Roach and Wulff 1987; Helenurm and Schaal 1996). To minimize these maternal effects, we first began by using seeds collected in the field to grow and pollinate a generation of parent plants under the same controlled conditions as above to produce standardized seeds for our experiment (for further details, see Brandenburger *et al.* 2019b). In October 2012, we planted 356 parent plants, of which 215 flowered and 186 produced seeds for use in our experiments.

### Seed size and germination

We calculated average seed mass by weighing all the air-dried seeds produced by each parent plant using a Mettler Toledo XS analytical balance, and then dividing by the number of seeds produced. We then randomly selected 10 seeds per Australian parent plant and 15 seeds per South African parent plant (unless there were not enough in which case, we used all available seeds) for germination and planting. In total, we had 1162 seeds from 111 parent plants. On the 3rd and 4th of December 2013, seeds from each parent plant were placed together in Petri dishes with moist filter paper and sealed with Parafilm. Petri dishes were checked every 3–4 days for germination, with any seedlings removed for planting. Ten weeks after first moistening the seeds, 91 % had germinated and 4 % had disintegrated. On the 10th of February 2014, we tested the remaining 5 % of the seeds (*n* = 55) for seed viability by slicing them open and staining them with a 0.1 % solution of tetrazolium chloride which stains respiring tissue pink (Hoyle 2022). This gave us the number of ungerminated but still viable seeds. Overall germination was calculated as (*germinated seeds*)/(*total seeds*). We also calculated germination of viable seeds: (*germinated seeds*)/(*germinated seeds* + *ungerminated but still viable seeds*).

### Reproductive output

**Figure 1.**
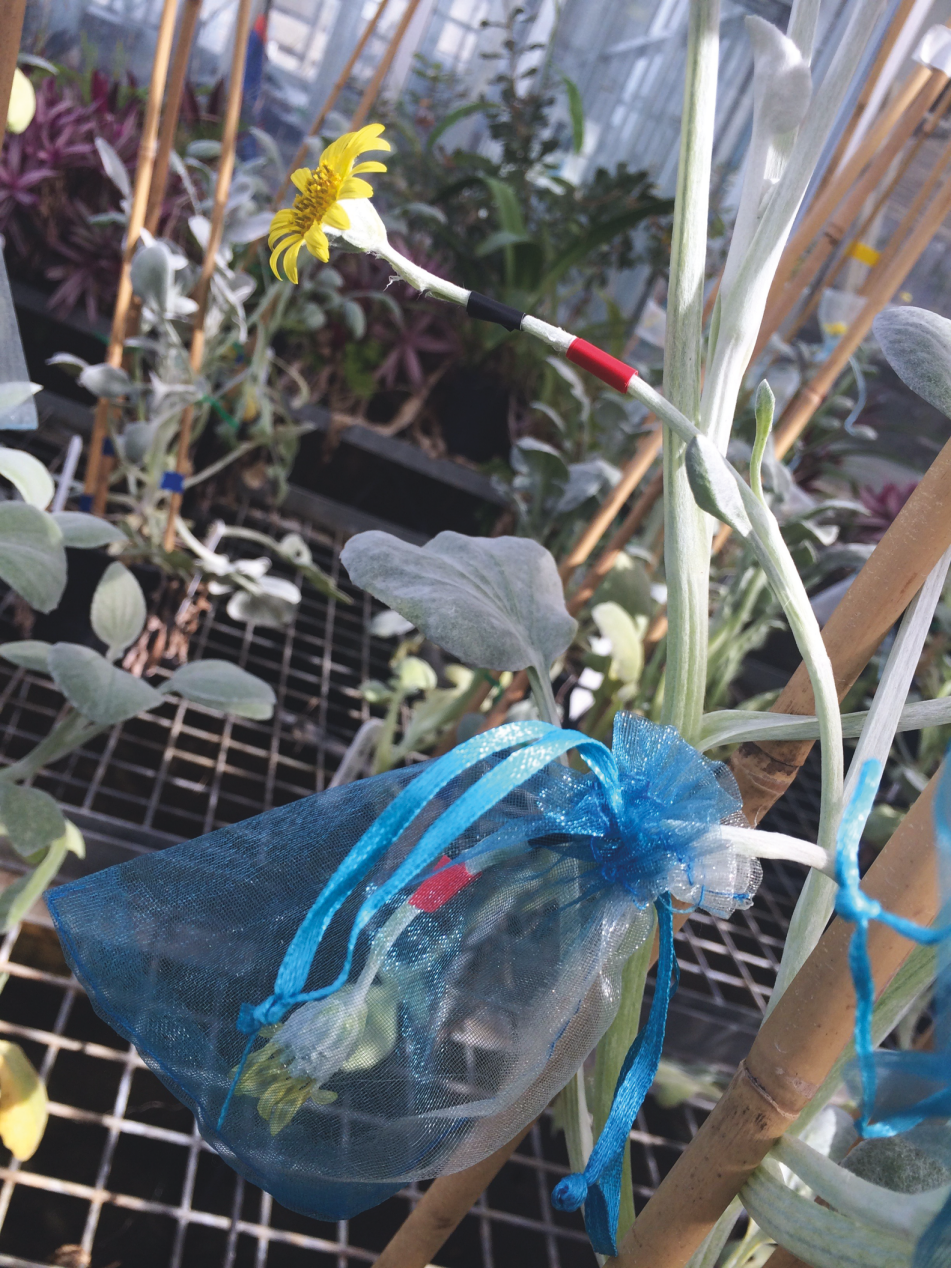
Illustration of pollination recording and seed collection techniques. Small strips of coloured electrical tape were used to tag inflorescences which had already been pollinated or counted, and small drawstring organza bags were used to exclude external pollinators and collect seeds.

We outcrossed all available individuals from each of the five populations (one South African and four Australian) every 3–4 days for the duration of the experiment. For each one of the five populations, we used a paintbrush to brush the pollen from all flowering individuals into a Petri dish. We then mixed the pollen and distributed it back to all the available inflorescences in that population. We used small strips of coloured electrical tape to tag inflorescences which had already been pollinated, and small drawstring organza bags to exclude external pollinators and collect seeds (Fig. 1). We ceased pollination on the 7th of October 2014 and finalized seed collection by the 30th of October 2014. To assess the allocation to reproductive biomass, we then selected a random subset of plants from each population, separated reproductive biomass from vegetative biomass and dried the material at 60 °C for 72 h before weighing it. For a standardized comparison of the proportion of biomass that plants were allocating to reproduction, we calculated reproductive biomass (%) as follows: *(reproductive biomass)/(total biomass)*.

To record what percentage of plants flowered, as well as the number of inflorescences produced by each plant, we grew another season of experimental plants between the 25th of November 2014 and the 26th of October 2015. We used seeds from the same batch as the previous season, and randomly selected five seeds per Australian parent plant and 10 seeds per South African parent plant (unless there were not enough in which case we used all available seeds) for germination and planting as before. In total, we had 401 seeds from 80 parent plants. Starting in February 2015 (before flowering began) and continuing until there was negligible flowering in both source and introduced plants (the end of May 2015), we checked the plants every 3–4 days and recorded whether a plant had flowered and how many inflorescences it had produced. We used small strips of coloured electrical tape to tag plants and inflorescences which had already been counted.

### Survival

To assess survival we recorded which plants survived until the harvest in November 2014, 11 months after the plants first germinated.

A summary of the experimental design **[see****Supporting Information—Table S1****]** and sample sizes for all variables and populations **[see****Supporting Information—Table S2****]** can be found in **Supporting Information**.

## Data analysis

Depending on the response variable, we analysed the data with generalized linear mixed models (glmm) or linear mixed models (lmm) using the ‘lme4’ package in R (Bates *et al.* 2015). We used the ‘multcomp’ package (Hothorn *et al.* 2008) to run a planned contrast between the one South African and four Australian populations, conditioning on family as a random effect. For *plants flowering* and *reproductive biomass*, singular fit and convergence errors were overcome by using the ‘glmmTMB’ package to create generalized linear mixed models built on the template model builder (Brooks *et al.* 2017). Likelihood ratio tests were then used to test for a ‘country’ effect whilst keeping the populations as random effects. *P*-values were adjusted using the Holm test (1979) to account for multiple hypothesis testing. All analyses were performed using R statistical software, version 3.5.2 (R Core Team 2019). For each variable, details regarding the data type, data family and the model we used can be found in **Supporting Information—Table S3**.

We checked for variation in the introduced range by comparing just the Australian populations according to each of the eight variables using one-way ANOVAs. Only two variables showed strong evidence towards a difference among Australian populations (Tables S4 and S5). This is in line with previous work showing that the majority of variables (29/30) show no significant differences among the four Australian populations (Brandenburger *et al.* 2019a, b, 2020; Table S6). Any differences among Australian populations are incorporated in the planned contrast between South African and Australian populations above. Adjusting *P*-values with a Holm adjustment (1979) did not change the significance outcomes in any of the eight one-way ANOVAs. (For full details, **see****Supporting Information—Appendix S1**).

## Results

**Figure 2.**
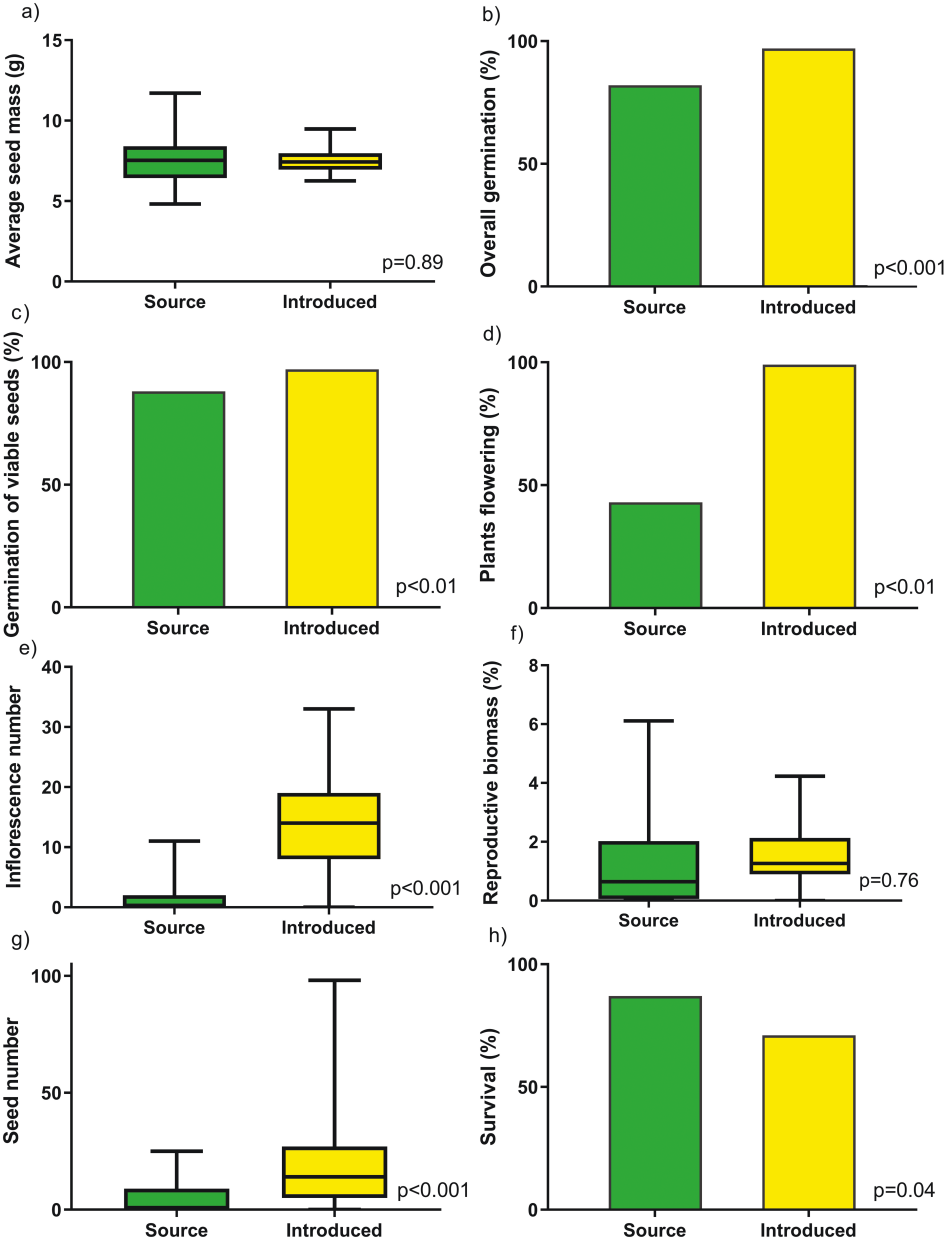
Life-history characteristics of South African source (green) and Australian introduced (yellow) *A. populifolia* plants. Boxes represent the 25th to 75th percentiles of data with whiskers extending from the 0 to 100 percentile; solid lines within the boxes indicate medians. (A) Average seed mass (g), (B) overall germination (%), (C) germination of viable seeds after 10 weeks (%), (D) number of plants flowering (%), (E) number of inflorescences produced per plant, (F) reproductive biomass (%), (G) number of seeds produced per plant, (H) survival until the end of the experiment (%).

Contrary to our expectations, we could not reject the null hypothesis of zero difference in average seed mass between source and introduced populations (*P* = 0.89; Fig. 2A). However, there were substantial differences in germination behaviour between the source and introduced populations. A higher percentage of the introduced seeds (97 %) germinated than did the source seeds (82 %) (*P* < 0.001, Fig. 2B). After 10 weeks, 99.5 % of the viable introduced seeds had germinated, but only 94.5 % of the viable source seeds had germinated, meaning that 5.5 % of the source seeds had been delayed for future germination (*P* < 0.01, Fig. 2C). Consistent with our hypotheses, we found strong evidence that the Australian introduced plants outperformed the South African source plants for most of the reproductive variables. Almost all introduced plants (99 %) produced inflorescences, while only 43 % of source plants flowered (*P* < 0.01, Fig. 2D). Introduced plants produced an average of 13 inflorescences per plant, while source plants only produced an average of 1.2 inflorescences per plant (*P* < 0.001, Fig. 2E). There was no evidence to suggest a difference between the reproductive biomass of the two groups (*P* = 0.76, Fig. 2F). With experimental outcrossing the introduced plants produced more than 10 times as many seeds as the source plants did (*P* < 0.001, Fig. 2G), a substantial relative fitness differential which demonstrates that the genotypic divergence is adaptive, not neutral. Only 74.1 % of the introduced plants survived to the end of the experiment compared with 86.5 % of the source plants (*P* = 0.04, Fig. 2H). Full model outputs including sample sizes and test statistics are shown in Table 1.

**Table 1.** Full model outputs for all variables showing: mean values for South African (SA) and Australian (AUS) plants; sample sizes (*n*) for SA and AUS plants; standard error for the mean difference between the two groups (SA and AUS); test statistics; adjusted *P*-values using the Holm correction (Holm 1979) to account for multiple hypothesis testing (no variables changed their significance using the Holm test). Likelihood ratio tests (LR) were undertaken for the glmmTMB models and planned contrasts (*z*) were undertaken for the other models.

Variable	Unit	Mean SA	Mean AUS	*n* SA	*n* AUS	Std error	Test statistic	Adj *P*-value
Average seed mass	(g)	7.56	7.59	21	89	0.225	*z* = 0.14	*P* = 0.89
Overall germination	(%)	82	97	272	890	0.390	*z* = 4.88	*P* < 0.001
Germination of viable seeds	(%)	94.5	99.5	243	870	0.731	*z* = 3.27	*P* < 0.01
Plants flowering	(%)	43	99	60	109	1.104	LR = 9.46	*P* < 0.01
Inflorescence number		1.2	13	60	109	0.167	*z* = 14.25	*P* < 0.001
Reproductive biomass	(% of total biomass)	0.012	0.015	36	41	0.200	LR = 0.77	*P* = 0.76
Seed number		0.8	9	39	89	0.478	*z* = 5.17	*P* < 0.001
Survival	(%)	86.6	74.2	119	217	0.331	*z* = −2.44	*P* = 0.04

## Discussion

In less than 90 years, Australian *A. populifolia* has evolved a suite of weedy and seedy life-history characteristics: the plants use a live-fast die-young strategy of germination and survival, and compared to the source population, the introduced plants produce more inflorescences and more seeds that germinate faster. These trends have been reported for several other introduced plants (Colautti *et al.* 2006; Parker *et al.* 2013; Yan *et al.* 2016; Burns *et al.* 2019), and may help to explain the spread and success of *A. populifolia* in Australia.

A recent review of species in their introduced and native ranges found a tendency for introduced plants to germinate earlier, at greater percentages and over a wider range of germination conditions than plants in the native range (Gioria and Pyšek 2017). Our results for *A. populifolia* are in line with this trend. Not only did the introduced plants have a higher per cent of germination compared to the source plants (Fig. 2B), but they also germinated nearly all (99.5 %) of their viable seeds within the first 10 weeks of the experiment, compared to the source plants which delayed 5.5 % of their seeds for future germination instead (Fig. 2C). The ‘bet-hedging’ strategy displayed by the source plants is usually attributed to plants needing to offset the risk of uncertain germination success by retaining some seeds for future germination (Venable 2007). The fact that introduced *A. populifolia* have evolved to germinate almost all of their viable seeds instead of retaining a portion for future germination is consistent with the idea that they are adapting to a less risky environment than is experienced by their source population. One possible explanation for this could be that the increased rainfall in the introduced range (throughout the year but especially in the hotter months; Brandenburger *et al.* 2019b) provides a less climatically risky environment, especially during germination when seedlings are likely to be more susceptible to desiccation. However, there may be other abiotic or biotic factors which could also contribute to a less risky environment in the introduced range (e.g. heightened reproductive success or decreased herbivory) which could possibly also contribute to the earlier emergence and success of introduced *A. populifolia* (Verdú and Traveset 2005). At the other end of the life cycle, introduced *A. populifolia* also displayed a faster life-history strategy, with significantly fewer Australian plants surviving until the end of the experiment compared with South African plants.

At nearly every stage of reproduction, introduced Australian *A. populifolia* outperformed source South African *A. populifolia* under these growing conditions. More plants produced flowers, each flowering plant produced approximately 10-fold as many flowers and seeds, and the resulting seeds germinated faster. Although the search for variables that were correlated with the success of introduced species has yielded idiosyncratic results over the last decades (Moles *et al.* 2012), in order for any species to survive and spread, it must be able to reproduce successfully. In addition, there is much evidence to support the idea that introduced species can develop an increase in reproductive output (Colautti *et al.* 2006; Hawkes 2007; Parker *et al.* 2013), with the tenets of the EICA hypothesis (fewer resources for defence in the introduced range can lead to more resources for growth and reproduction; Blossey and Notzold 1995) often underpinning these results. Introduced plants must then allocate these resources in a trade-off between sexual and vegetative reproduction for the greatest chance of successful establishment in a new range (Sakai *et al.* 2001).

We predicted that enemy release (Keane and Crawley 2002) may allow introduced *A. populifolia* to allocate more energy to seed production and thus produce larger seeds. The fact that introduced *A. populifolia* produced substantially more seeds than did native *A. populifolia* is consistent with the idea that the introduced population might have more energy available for reproduction. However, in this case, it appears that selection favoured the production of more seeds rather than the production of larger seeds (Fig. 2A and G). Similar increases in seed number rather than seed size have been observed in other studies, e.g. in *Pinus taeda* grown under elevated CO_2_ (Way *et al.* 2010). However, there are also examples of species increasing seed mass rather than number when resource availability is higher (see Venable 1992). The differing results for different species likely reflect the fact that a species’ seed mass is the result of a complex interplay of trade-offs and selective pressures related to seed production, seed predation, seed dispersal, survival in soil, germination success and seedling establishment (Moles and Westoby 2006). In short, it is not so simple as larger seeds being better than small ones.

In the case of *A. populifolia*, we found that even though the introduced plants produce 10 times as many flowers as source plants, there was no difference between the reproductive biomass produced by each group. One explanation in this case would be that introduced *A. populifolia* have evolved to produce more, smaller flowers using the same amount of available floral resources (Sargent *et al.* 2007). This in turn would have implications for pollination and seed-set and could be an interesting area for future research. Another possible explanation is that the very low variation in the introduced range (Rollins *et al.* 2013) might have resulted in a lack of suitable allelic variation at the relevant locus to change total reproductive biomass. In addition, it is not uncommon for related characteristics to have negative genetic correlations, for example leaf thickness versus leaf number (~total leaf biomass) (Etterson and Shaw 2001). With low variation in the introduced population, breaking out of such a negative correlation would be unlikely. Interestingly, Rollins *et al.* (2013) showed that although many species of introduced populations which showed adaptation retained 81 % of their genetic diversity, others can establish and spread successfully even with very low levels of genetic diversity. In particular, the introduced populations of *A. populifolia* in our study only retained ≤3 % of their native diversity (*H*_e_) after introduction—the lowest level out of the 36 species that they investigated.

Even though germination, reproduction and survival are basic elements of a plant’s life history, this is the first study to our knowledge that compares the life history of an introduced plant species with its original source population. Instead of using a broad range of home range populations, we used genetic techniques to pinpoint the single-parent population for this introduction to provide a precise and powerful assessment of evolutionary divergence. Our study adds to the remarkable data that we already have on the rapid evolutionary changes occurring in the morphology (Brandenburger *et al.* 2019b), physiology (Brandenburger *et al.* 2019a) and defence traits (Brandenburger *et al.* 2020) of introduced *A. populifolia*. This species now joins a host of other introduced plants and animals in both terrestrial and marine environments undergoing dramatic changes—including the cane toad *Rhinella marina* (Phillips *et al.* 2006; Phillips and Shine 2006), the marine copepod *Eurytemora affinis* (Lee 1999) and the brown seaweed *Fucus evanescens* (Wikström *et al.* 2006).

With evidence that introduced species can still undergo morphological changes more than a century after introduction (Flores-Moreno *et al.* 2015), we can expect that some introduced species will continue to change over time as a result of various ecological–evolutionary interactions (Lambrinos 2004), including drivers such as rapid environmental change or an evolutionary arms race between introduced plants and their competitors or herbivores. Unfortunately, it appears that policy and conservation biology are only just beginning to take this into account: more than half of plant invasion impact studies have lasted less than a year, with less than a 10th being conducted over 4 or more years (Stricker *et al.* 2015). If we are to fully understand and manage introduced species, then we must consider the speed and scope of their potential for evolutionary change (Stockwell *et al.* 2003; Smith and Bernatchez 2008).

## Supporting Information

The following additional information is available in the online version of this article—

Table S1. A summary of the experimental design details.

Table S2. Sample sizes for each population for each variable.

Table S3. Data type, data family and model used for each plant variable.

Appendix S1. Latitudinal variation in Australia.

Table S4. A comparison of each variable among only the four introduced populations in Australia.

Table S5. Mean values for the two variables showing differences among Australian populations.

Table S6. Results of one-way ANOVAs from previous studies contrasting each variable among only the four introduced populations in Australia.

plac038_suppl_Supplementary_Material

## Data Availability

Data and code are available on the Dryad Digital Repository (https://datadryad.org/) under the following doi:10.5061/dryad.wh70rxwqx.

## Notes

Evolution & Diversity. Chief Editor: Jeremy Beaulieu

## References

[CIT0001] AVH database. 2022. *Australia’s virtual herbarium.* Council of Heads of Australasian Herbaria. http://avh.ala.org.au (1 June 2022).

[CIT0002] Baker HG. 1965. Characteristics and modes of origin of weeds. In: Baker HG, Stebbins GL, eds. The genetics of colonising species, 1st edn. New York: Academic Press, 147–168.

[CIT0003] Baker HG. 1974. The evolution of weeds. Annual Review of Ecology and Systematics 5:1–24.

[CIT0004] Bates D , Machler M, Bolker BM, Walker SC. 2015. Fitting linear mixed-effects models using lme4. Journal of Statistical Software 67:1–48.

[CIT0005] Blair AC , Wolfe LM. 2004. The evolution of an invasive plant: an experimental study with *Silene latifolia*. Ecology 85:3035–3042.

[CIT0006] Blossey B , Notzold R. 1995. Evolution of increased competitive ability in invasive nonindigenous plants: a hypothesis. Journal of Ecology 83:887–889.

[CIT0007] Bossdorf O , Auge H, Lafuma L, Rogers WE, Siemann E, Prati D. 2005. Phenotypic and genetic differentiation between native and introduced plant populations. Oecologia 144:1–11. 15891837 10.1007/s00442-005-0070-z

[CIT0008] Brandenburger CR , Cooke J, Sherwin WB, Moles AT. 2019a. Rapid evolution of leaf physiology in an introduced beach daisy. Proceedings of the Royal Society B 286:20191103. 31455190 10.1098/rspb.2019.1103 PMC6732393

[CIT0009] Brandenburger CR , Kim M, Slavich E, Meredith FL, Salminen JP, Sherwin WB, Moles AT. 2020. Evolution of defense and herbivory in introduced plants—testing enemy release using a known source population, herbivore trials, and time since introduction. Ecology and Evolution 10:5451–5463. 32607166 10.1002/ece3.6288 PMC7319247

[CIT0010] Brandenburger CR , Sherwin WB, Creer SM, Buitenwerf R, Poore AG, Frankham R, Finnerty PB, Moles AT. 2019b. Rapid reshaping: the evolution of morphological changes in an introduced beach daisy. Proceedings of the Royal Society B 286:20181713. 30963824 10.1098/rspb.2018.1713 PMC6408894

[CIT0011] Brooks ME , Kristensen K, van Benthem KJ, Magnusson A, Berg CW, Nielsen A, Skaug HJ, Machler M, Bolker BM. 2017. glmmTMB balances speed and flexibility among packages for zero-inflated generalized linear mixed modeling. The R Journal 9:378–400.

[CIT0012] Burns JH , Bennett JM, Li J, Xia J, Arceo-Gómez G, Burd M, Burkle LA, Durka W, Ellis AG, Freitas L. 2019. Plant traits moderate pollen limitation of introduced and native plants: a phylogenetic meta-analysis of global scale. New Phytologist 223:2063–2075. 31116447 10.1111/nph.15935

[CIT0013] Buswell JM , Moles AT, Hartley S. 2011. Is rapid evolution common in introduced pant species? Journal of Ecology 99:214–224.

[CIT0014] Colautti RI , Grigorovich IA, MacIsaac HJ. 2006. Propagule pressure: a null model for biological invasions. Biological Invasions 8:1023–1037.

[CIT0015] Colautti RI , Maron JL, Barrett SCH. 2009. Common garden comparisons of native and introduced plant populations: latitudinal clines can obscure evolutionary inferences. Evolutionary Applications 2:187–199. 25567860 10.1111/j.1752-4571.2008.00053.x PMC3352372

[CIT0016] Crawley MJ. 1997. Plant ecology, 2nd edn. Cambridge: Wiley-Blackwell.

[CIT0017] Daehler CC , Strong DR. 1997. Reduced herbivore resistance in introduced smooth cordgrass (*Spartina alterniflora*) after a century of herbivore-free growth. Oecologia 110:99–108. 28307474 10.1007/s004420050138

[CIT0018] Daws M , Hall J, Flynn S, Pritchard H. 2007. Do invasive species have bigger seeds? Evidence from intra- and inter-specific comparisons. South African Journal of Botany 73:138–143.

[CIT0019] Etterson JR , Shaw RG. 2001. Constraint to adaptive evolution in response to global warming. Science 294:151–154. 11588260 10.1126/science.1063656

[CIT0020] Felker-Quinn E , Schweitzer JA, Bailey JK. 2013. Meta-analysis reveals evolution in invasive plant species but little support for evolution of increased competitive ability (EICA). Ecology and Evolution 3:739–751. 23531703 10.1002/ece3.488 PMC3605860

[CIT0021] Flores-Moreno H , García-Treviño ES, Letten AD, Moles AT. 2015. In the beginning: phenotypic change in three invasive species through their first two centuries since introduction. Biological Invasions 17:1215–1225.

[CIT0022] Gioria M , Pyšek P. 2017. Early bird catches the worm: germination as a critical step in plant invasion. Biological Invasions 19:1055–1080.

[CIT0023] Harper JL , Lovell P, Moore K. 1970. The shapes and sizes of seeds. Annual Review of Ecology and Systematics 1:327–356.

[CIT0024] Hawkes CV. 2007. Are invaders moving targets? The generality and persistence of advantages in size, reproduction, and enemy release in invasive plant species with time since introduction. American Naturalist 170:832–843. 10.1086/522842 18171166

[CIT0025] Helenurm K , Schaal BA. 1996. Genetic and maternal effects on offspring fitness in *Lupinus texensis* (Fabaceae). American Journal of Botany 83:1596–1608.

[CIT0026] Hesp PA. 1991. Ecological processes and plant adaptations on coastal dunes. Journal of Arid Environments 21:165–191.

[CIT0027] Hierro JL , Eren O, Khetsuriani L, Diaconu A, Török K, Montesinos D, Andonian K, Kikodze D, Janoian L, Villarreal D. 2009. Germination responses of an invasive species in native and non-native ranges. Oikos 118:529–538.

[CIT0028] Holm S. 1979. A simple sequentially rejective multiple test procedure. Scandinavian Journal of Statistics 6:65–70.

[CIT0029] Hothorn T , Bretz F, Westfall P. 2008. Simultaneous inference in general parametric models. Biometrical Journal 50:346–363. 18481363 10.1002/bimj.200810425

[CIT0030] Hoyle G. 2022. *Seed viability, TZ testing.* https://prometheusprotocols.net (21 August 2022).

[CIT0031] Jakobsson A , Eriksson O. 2000. A comparative study of seed number, seed size, seedling size and recruitment in grassland plants. Oikos 88:494–502.

[CIT0032] Keane RM , Crawley MJ. 2002. Exotic plant invasions and the enemy release hypothesis. Trends in Ecology and Evolution 17:164–170.

[CIT0033] Lambrinos JG. 2004. How interactions between ecology and evolution influence contemporary invasion dynamics. Ecology 85:2061–2070.

[CIT0034] Lee CE. 1999. Rapid and repeated invasions of fresh water by the copepod *Eurytemora affinis*. Evolution 53:1423–1434. 28565555 10.1111/j.1558-5646.1999.tb05407.x

[CIT0035] Lee CE. 2002. Evolutionary genetics of invasive species. Trends in Ecology & Evolution 17:386–391.

[CIT0036] Leishman MR , Wright IJ, Moles AT, Westoby M. 2000. The evolutionary ecology of seed size. In: Fenner M, ed. Seeds: the ecology of regeneration in plant communities, 2nd edn. Wallingford, UK: CABI Publishing, 31–57.

[CIT0037] Maron JL , Vilà M, Bommarco R, Elmendorf S, Beardsley P. 2004. Rapid evolution of an invasive plant. Ecological Monographs 74:261–280.

[CIT0038] Mason RA , Cooke J, Moles AT, Leishman MR. 2008. Reproductive output of invasive versus native plants. Global Ecology and Biogeography 17:633–640.

[CIT0039] Moles AT , Flores-Moreno H, Bonser SP, Warton DI, Helm A, Warman L, Eldridge DJ, Jurado E, Hemmings FA, Reich PB. 2012. Invasions: the trail behind, the path ahead, and a test of a disturbing idea. Journal of Ecology 100:116–127.

[CIT0040] Moles AT , Leishman MR. 2008. The seedling as part of a plant’s life history strategy. In: Leck MA, Parker VT, Simpson RL, eds. Seedling ecology and evolution. Cambridge, UK: University Press, 217–238.

[CIT0041] Moles AT , Westoby M. 2006. Seed size and plant strategy across the whole life cycle. Oikos 113:91–105.

[CIT0042] Parker JD , Torchin ME, Hufbauer RA, Lemoine NP, Alba C, Blumenthal DM, Bossdorf O, Byers JE, Dunn AM, Heckman RW. 2013. Do invasive species perform better in their new ranges? Ecology 94:985–994. 23858639 10.1890/12-1810.1

[CIT0043] Phillips BL , Brown GP, Webb JK, Shine R. 2006. Invasion and the evolution of speed in toads. Nature 439:803. 16482148 10.1038/439803a

[CIT0044] Phillips BL , Shine R. 2006. An invasive species induces rapid adaptive change in a native predator: cane toads and black snakes in Australia. Proceedings of the Royal Society B: Biological Sciences 273:1545–1550. 10.1098/rspb.2006.3479 PMC1560325 16777750

[CIT0045] Pigliucci M. 2001. Phenotypic plasticity: beyond nature and nurture, 1st edn. Baltimore, MD: JHU Press.

[CIT0046] R Core Team. 2019. *R: a language and environment for statistical computing.* Version *3.5.1*. Vienna, Austria: R Foundation for Statistical Computing.

[CIT0047] Roach DA , Wulff RD. 1987. Maternal effects in plants. Annual Review of Ecology and Systematics 18:209–235.

[CIT0048] Rollins LA , Moles AT, Lam S, Buitenwerf R, Buswell JM, Brandenburger CR, Flores-Moreno H, Nielsen KB, Couchman E, Brown GS, Thomson FJ, Hemmings F, Frankham R, Sherwin WB. 2013. High genetic diversity is not essential for successful introduction. Ecology and Evolution 3:4501–4517. 24340190 10.1002/ece3.824 PMC3856749

[CIT0049] Sakai AK , Allendorf FW, Holt JS, Lodge DM, Molofsky J, With KA, Baughman S, Cabin RJ, Cohen JE, Ellstrand NC, McCauley DE, O’Neil P, Parker IM, Thompson JN, Weller SG. 2001. The population biology of invasive species. Annual Review of Ecology and Systematics 32:305–332.

[CIT0050] SANBI Database. 2017. *South African National Biodiversity Institute*. http://pza.sanbi.org (20 June 2017).

[CIT0051] Sargent RD , Goodwillie C, Kalisz S, Ree RH. 2007. Phylogenetic evidence for a flower size and number trade-off. American Journal of Botany 94:2059–2062. 21636399 10.3732/ajb.94.12.2059

[CIT0052] Smith TB , Bernatchez L. 2008. Evolutionary change in human-altered environments. Molecular Ecology 17:1–8. 18173497 10.1111/j.1365-294X.2007.03607.x

[CIT0053] Stastny M , Schaffner U, Elle E. 2005. Do vigour of introduced populations and escape from specialist herbivores contribute to invasiveness? Journal of Ecology 93:27–37.

[CIT0054] Stockwell CA , Hendry AP, Kinnison MT. 2003. Contemporary evolution meets conservation biology. Trends in Ecology & Evolution 18:94–101.

[CIT0055] Stricker KB , Hagan D, Flory SL. 2015. Improving methods to evaluate the impacts of plant invasions: lessons from 40 years of research. AoB Plants 7:plv028; doi:10.1093/aobpla/plv028. 25829379 PMC4418169

[CIT0056] van Kleunen M , Weber E, Fischer M. 2010. A meta-analysis of trait differences between invasive and non-invasive plant species. Ecology Letters 13:235–245. 20002494 10.1111/j.1461-0248.2009.01418.x

[CIT0057] Venable DL. 1992. Size-number trade-offs and the variation of seed size with plant resource status. The American Naturalist 140:287–304.

[CIT0058] Venable DL. 2007. Bet hedging in a guild of desert annuals. Ecology 88:1086–1090. 17536393 10.1890/06-1495

[CIT0059] Verdú M , Traveset A. 2005. Early emergence enhances plant fitness: a phylogenetically controlled meta-analysis. Ecology 86:1385–1394.

[CIT0060] Violle C , Navas ML, Vile D, Kazakou E, Fortunel C, Hummel I, Garnier E. 2007. Let the concept of trait be functional! Oikos 116:882–892.

[CIT0061] Way DA , Ladeau SL, McCarthy HR, Clark JS, Oren R, Finzi AC, Jackson RB. 2010. Greater seed production in elevated CO_2_ is not accompanied by reduced seed quality in *Pinus taeda* L. Global Change Biology 16:1046–1056.

[CIT0062] Wikström SA , Steinarsdóttir MB, Kautsky L, Pavia H. 2006. Increased chemical resistance explains low herbivore colonization of introduced seaweed. Oecologia 148:593–601. 16532333 10.1007/s00442-006-0407-2

[CIT0063] Willis AJ , Blossey B. 1999. Benign environments do not explain the increased vigour of non-indigenous plants: a cross-continental transplant experiment. Biocontrol Science and Technology 9:567–577.

[CIT0064] Yan XH , Zhou B, Yin ZF, Wang N, Zhang ZG. 2016. Reproductive biological characteristics potentially contributed to invasiveness in an alien invasive plant *Bidens frondosa*. Plant Species Biology 31:107–116.

