# Supplementary material for: Weedy and seedy: the rapid evolution of life-history characteristics in an introduced daisy
Source: AoB Plants. 2022 Aug 18;14(5):plac038. doi: 10.1093/aobpla/plac038 (PMC9449359; doi:10.1093/aobpla/plac038)
Supplement: plac038_suppl_Supplementary_Material [file plac038_suppl_supplementary_material.pdf]

SUPPORTING INFORMATION

Weedy and seedy: the rapid evolution of life-history characteristics in an introduced daisy

Claire R. Brandenburger*[1], Ben Maslen[2], William B. Sherwin[1] and Angela T. Moles[1]

[1] Evolution & Ecology Research Centre, School of Biological, Earth and Environmental Sciences, UNSW Sydney NSW 2052, Australia.

[2] Mark Wainwright Analytical Centre, UNSW Sydney NSW 2052, Australia.

*Author for correspondence. Email: claire.brandenburger@unsw.edu.au

Table S1. A summary of the experimental design details.

Table S2. Sample sizes for each population for each variable.

Table S3. Data type, data family and model used for each plant variable.

Appendix S1. Latitudinal variation in Australia.

Table S4. Results of one-way ANOVAs from previous studies contrasting each variable among only the four introduced populations in Australia.

Table S5. A comparison of each variable among only the four introduced populations in Australia.

Table S6. Mean values for the two variables showing differences among Australian populations.

Table S1. A summary of the experimental design details. Dates for the beginning and end of each experimental season are given, along with the variables measured in each season. All plants were grown under controlled glasshouse temperatures, daily watering with automatic drippers at 5 pm, and the same soil composition.

**Season of germination**	Spring 2012	Spring 2013	Spring 2014
**Date water added to seeds**	21, 22 Oct 2012	3, 4 Dec 2013	25 Nov 2014 (163 seeds) and 11 Dec 2014 (six seeds which we overlooked)*
**Experiment end date**	NA	30 Oct 2014	26 Oct 2015
**Variables measured**	This season we grew a generation of parent plants to provide standardised seeds which we then used for the following two experimental seasons.	• Average seed mass<br>• Overall germination<br>• Germination of viable seeds<br>• Reproductive biomass<br>• Seed number<br>• Survival	• Plants flowering<br>• Inflorescence number

*Although the six seeds had water added 16 days after the other 163 seeds, they were only planted five to six days later.

Table S2. Sample sizes for each population for each variable.

**Variable**	**Arniston (SA)**	**Treachery Beach (AUS)**	**Mallacoota (AUS)**	**Narooma (AUS)**	**Wairo Beach (AUS)**
Average seed mass	21	13	33	12	31
Overall germination	272	130	330	120	310
Germination of viable seeds	243	126	323	117	304
Plants flowering	60	26	29	24	30
Inflorescence number	60	26	29	24	30
Reproductive biomass	36	11	12	7	11
Seed number	39	13	33	12	31
Survival	119	40	70	39	68

Table S3. Data type, data family and model used for each plant variable. Model types: lmm = linear mixed model, glmm = generalised linear mixed model, glmmTMB = generalised linear mixed model built on the template model builder.

Variable	Data type	Data family	Model
Average seed mass	Continuous	Normal	lm
Overall germination	Binary	Binomial	glmm
Germination of viable seeds	Binary	Binomial	glmm
Plants flowering	Binary	Binomial	glmmTMB
Inflorescence number	Discrete	Poisson	glmm
Reproductive biomass	0-100%	Tweedie	glmmTMB
Seed number	Discrete	Poisson	glmm
Survival	Binary	Binomial	glmm

Appendix S1: Latitudinal variation in Australia

Previous work (Brandenburger *et al.*, 2019a, Brandenburger *et al.*, 2019b, Brandenburger *et al.*, 2020) has shown that 29/30 variables do not differ among the four Australian populations. In this study we were surprised to find that for two of the eight life-history variables, there was a significant difference among Australian populations (Table S4). In both cases, the northernmost population at Treachery Beach was significantly different from the other three Australian populations. The Treachery Beach population produced seeds that were approximately 12% bigger than the other populations and had an average of 93% plants survive to the end of the experiment compared to only 64% of plants from the other populations (Table S5). This is an interesting outcome and would merit further investigation. Details of the 30 variables from previous studies are listed here for convenience in Table S6.

Table S4. A comparison of each variable among only the four introduced populations in Australia. P-values and test statistics are the result of one-way analyses of variance (ANOVAs). Post-hoc pairwise tests were then undergone for variables that showed strong evidence of a difference through the ANOVAs. The p-values from the ANOVAs were adjusted using a Holm adjustment (Holm, 1979) to account for multiple hypothesis testing.

Variable	p-value	Test statistic	Significant pairwise comparisons	Adj p-value
Average seed mass	p<0.001	9.86	Treachery versus:<br>• Mallacoota p<0.001<br>• Narooma p=0.01<br>• Wairo p<0.001	p<0.001
Overall germination	0.17	5.04	NA	1
Germination of viable seeds	0.72	1.34	NA	1
Plants flowering	0.41	2.90	NA	1
Inflorescence number	0.18	4.85	NA	1
Reproductive biomass	0.41	2.91	NA	1
Seed number	0.32	3.52	NA	1
Survival	p<0.01	14.73	Treachery versus:<br>• Mallacoota p=0.01<br>• Narooma p=0.02<br>• Wairo p=0.01	p=0.014

Table S5. Mean values for the two variables showing differences among Australian populations. Populations are shown in order of latitude with Treachery Beach being the northernmost population.

Population	Seed mass (g)	Survival (%)
**Treachery Beach**	8.27	92.5
**Wairo Beach**	7.20	63.2
**Narooma**	7.43	64.1
**Mallacoota**	7.47	64.3

Table S6. Results of one-way analyses of variance (ANOVAs) from previous studies contrasting each variable among only the four introduced populations in Australia. The p-values shown here from Brandenburger et al. 2019a and 2019b are those obtained after using a Holm adjustment (Holm 1979) to account for multiple tests undertaken in those studies.

**Variable**	**MS**	**F**	**p-value**	**Signif outcome**	**Reference paper**
Leaf dry matter content	497.0	7.247	0.013	SIG	Brandenburger et.al 2019b
Leaf density	515.7	3.265	0.264	NON SIG	Brandenburger et.al 2019b
Leaf thickness	0.028	2.825	0.440	NON SIG	Brandenburger et.al 2019b
Plant growth form	0.051	2.714	0.460	NON SIG	Brandenburger et.al 2019b
Leaf shape	0.094	2.680	0.460	NON SIG	Brandenburger et.al 2019b
Plant length	13651	2.526	0.472	NON SIG	Brandenburger et.al 2019b
$_{log10}$(Specific leaf area)	0.015	2.410	0.476	NON SIG	Brandenburger et.al 2019b
Leaf area	105.5	2.087	0.618	NON SIG	Brandenburger et.al 2019b
Plant height	3231	1.329	1.000	NON SIG	Brandenburger et.al 2019b
Above-ground biomass at 11 months	49.99	0.641	1.000	NON SIG	Brandenburger et.al 2019b
Below-ground biomass at 12 weeks	0.019	0.568	1.000	NON SIG	Brandenburger et.al 2019b
Total biomass at 12 weeks	0.114	0.480	1.000	NON SIG	Brandenburger et.al 2019b
Above-ground biomass at 12 weeks	0.044	0.362	1.000	NON SIG	Brandenburger et.al 2019b
Stomatal density on bottom of leaf	3338	4.207	0.156	NON SIG	Brandenburger et.al 2019a
Hair density on top of leaf	192.4	2.581	0.792	NON SIG	Brandenburger et.al 2019a
Photosynthetic nitrogen-use efficiency	10.52	1.883	1.000	NON SIG	Brandenburger et.al 2019a
Nitrogen per leaf area	0.284	1.689	1.000	NON SIG	Brandenburger et.al 2019a

Stomatal density on top of leaf	2952	1.554	1.000	NON SIG	Brandenburger et.al 2019a
Maximum rate of carboxylation (V_{cmax})	119.0	0.528	1.000	NON SIG	Brandenburger et.al 2019a
Intercellular CO_2 (Ci)	123.3	0.489	1.000	NON SIG	Brandenburger et.al 2019a
Maximum rate of electron transport (J_{max})	220.6	0.446	1.000	NON SIG	Brandenburger et.al 2019a
Hair density on bottom of leaf	194.0	0.434	1.000	NON SIG	Brandenburger et.al 2019a
Water-use efficiency	42.53	0.336	1.000	NON SIG	Brandenburger et.al 2019a
CO_2 assimilation rate (A_{area})	4.574	0.312	1.000	NON SIG	Brandenburger et.al 2019a
Stomatal conductance (g_s)	0.015	0.237	1.000	NON SIG	Brandenburger et.al 2019a
Ash	NA	0.21	0.89	NON SIG	Brandenburger et al. 2020
Alkaloids	NA	1.80	0.18	NON SIG	Brandenburger et al. 2020
C:N ratio	NA	1.47	0.25	NON SIG	Brandenburger et al. 2020
Phenols	NA	0.90	0.47	NON SIG	Brandenburger et al. 2020
Toughness	NA	1.01	0.39	NON SIG	Brandenburger et al. 2020

References

Brandenburger CR, Cooke J, Sherwin WB, Moles AT. 2019a. Rapid evolution of leaf physiology in an introduced beach daisy. *Proceedings of the Royal Society B,* 286:20191103.

Brandenburger CR, Kim M, Slavich E, Meredith FL, Salminen JP, Sherwin WB, Moles AT. 2020. Evolution of defense and herbivory in introduced plants—Testing enemy release using a known source population, herbivore trials, and time since introduction. *Ecology and Evolution,* 10:5451-5463.

Brandenburger CR, Sherwin WB, Creer SM, Buitenwerf R, Poore AG, Frankham R, Finnerty PB, Moles AT. 2019b. Rapid reshaping: the evolution of morphological changes in an introduced beach daisy. *Proceedings of the Royal Society B,* 286:20181713.

Holm S. 1979. A simple sequentially rejective multiple test procedure. *Scandinavian Journal of Statistics,* 6:65-70.
